# Covid-19—The real role of NSAIDs in Italy

**DOI:** 10.1186/s13018-020-01682-x

**Published:** 2020-05-04

**Authors:** Laura de Girolamo, Giuseppe M. Peretti, Nicola Maffulli, Anna T. Brini

**Affiliations:** 1 IRCCS Istituto Ortopedico Galeazzi, Via R. Galeazzi 4, 20161 Milan, Italy; 2 Department of Biomedical Sciences for Health, University of Milan, Via Mangiagalli 31, 20133 Milan, Italy; 3 Department of Medicine, Surgery and Dentistry, University of Salerno, Via S. Allende, 84081 Baronissi, SA Italy; 4 Barts and the London School of Medicine and Dentistry, Centre for Sports and Exercise Medicine, Mile End Hospital, Queen Mary University of London, 275 Bancroft Road, London, E1 4DG England; 5 School of Pharmacy and Bioengineering, Keele University School of Medicine, Thornburrow Drive, Stoke on Trent, England; 6 Department of Biomedical, Surgical and Dental Sciences, University of Milan, Via Vanvitelli 32, 20129 Milan, Italy

On behalf of the scientists and surgeons of one of the biggest orthopedic institutes in Italy, located in Milan, we feel the urge to share our position about the recent warnings on the use of anti-inflammatory drugs (NSAIDs) in this coronavirus “era.” Similarly to the antihypertensive drugs angiotensin-converting enzyme (ACE) inhibitors, angiotensin receptor blockers (ARBs), and beta blockers, and the oral antidiabetic drugs thiazolidinediones, some author have suggested that NSAIDs, particularly Ibuprofen, may induce increased sensitivity to more severe clinical features in coronavirus (Covid-19) infection.

NSAIDs are widely used worldwide. In musculoskeletal medicine, they keep under control osteoarthritis, tendinopathies, bursitis, muscle soreness, radicular pain, and others. In Italy, about 12 millions of adults have osteoarthritis or rheumatoid arthritis, a number estimated to grow substantially in the next years [1]. Moreover, an extensive increase of orthopedic surgeries in both developed and developing countries, the low cost of drugs, and the progressive rise of the geriatric population have contributed to the growth of global NSAIDs use. According to the Italian Medicines Agency (AIFA), in 2018 the defined daily doses (DDDs) per 1000 inhabitants per day was 18.6 [2].

The warning about the possible role of NSAIDs in worsening the severity of Covid-19 infection was very powerful because it was spread by a representative of the national medical authority of one of the European countries strongly interested by this pandemic. The warning also suggested to the population already under anti-inflammatory drugs to ask their general practitioners for advice, unleashing a wave of panic among the population, especially through social media. Other official communications by the national health systems and emergency management agency (EMA) [3] attempted to bring back calm and rationality, with poor results.

The effects of this warning has been very deleterious on our patients, especially on those who need to use these drugs for long periods for their medical conditions: NSAIDs allow these patients to lead a good quality of life, although we acknowledge that the prolonged use of these drugs may cause side effects, including kidney failure, liver failure, gastric ulcers, asthma attacks, heart and circulatory problems, and a partial immune depression [4].

Being based in Milan, we, more than others, are directly experiencing the devastating effects of this pandemic. We have been ready, and we still are now, to take any useful measures to reduce its impact on our population, without underestimating any possible new scientific information. Nevertheless, albeit in the midst of a general intellectual and emotional confusion, we try to maintain our scientific judgment, and would like to underline the danger of unproven and unfounded information, especially when spread out through mass and social media, which directly reach the general population without any filter.

The current body of scientific literature reports just a few articles analyzing the relationship between exposure to NSAIDs and possible complications in patients with pneumonia. A recent article investigated whether exposure to NSAIDs prior to hospital admission among patients suffering from community-acquired pneumonia (CAP) was associated with the development of pleural complications or a lung abscess. NSAIDs intake was independently associated with the development of pleuroparenchymal complications (odds ratio = 2.57 [1.02–6.64], *p* = 0.049). Nevertheless, given the lack of consistent results about the direct effect of NSAIDs on the immunological response and the lack of controlled randomized studies in human, the authors could not conclude a direct effect of NSAIDs in the development of the complications of CAP [5]. Surely, in addition to the supposed interfering effect of NSAIDs with responses mediated by the prostaglandin and leukotriene pathways, NSAIDs, together with other antipyretic and analgesic medications, can be responsible for later presentation of symptoms or underestimation of the severity of the disease, both leading to a delayed diagnosis and possible worse prognosis. A particular role in exacerbating the Covid-19 infection seems to have been assigned to Ibuprofen [6]. In diabetic rats, this chemical has induced an over-expression of ACE2 that we now know might be associated, theoretically, to an increase in susceptibility to Covid-19 infection [7]. However, the original purpose of the study was different, and this observation should therefore be verified in a more specific ad hoc experimental model. In addition, it is unclear how this study in animals with a metabolic disorder would translate to human medicine.

Despite the common global interest in preventing our patients from suffering the most serious consequences of this new infection, it would be dangerous to stop some of these drugs based on not confirmed data. The effects of such suspension could certainly worsen the conditions of some patients, adding a supplementary burden on our health providers who are already overwhelmed by this enormous number of serious Covid-19 cases.

Physicians may consider to replace the molecule indicated to be “most vile,” Ibuprofen, with a similar one while waiting for more definitive data. In general, and not only in these pandemic times, we agree that NSAIDs should be taken only when strictly necessary, at the lowest effective dose for the shortest possible period, possibly under a medical guidance and not as a self-medication. Therefore, when indicated, we are suggesting that our patients use drugs with less severe adverse effects, such as paracetamol (i.e. acetaminophen), pointing in any case out that, in this period, other NSAIDs and paracetamol could also mask the pyrexial signs of COVID-19, resulting in a delayed diagnosis of the infection.

The risks deriving from the use of NSAIDS and the Covid-19 infection should be analyzed from two different perspectives: (i) risks related to exposure to NSAIDs before the patient has been infected and (ii) risks related to the use of a drug during the COVID-19 infection. These two scenarios are dramatically different, and pose different scientific questions, which should be addressed by adequate studies. In other words, would the administration of this drug before being infected causes the same effect of taking it during the infection period? We also have to distinguish between the possible increase in susceptibility to the virus, especially in terms of severity, and the increase in aberrant host response.

Patients continue to produce the virus for weeks: the treatment of infected patients with immunosuppressant such as corticosteroids might increase the amount of virus replication [8]. Therefore, we certainly agree with interrupting any administration of immunosuppressant drugs in patients with confirmed or suspect diagnosis of Covid-19 infection. Further, given the concerns of a link between NSAIDs and both respiratory and cardiovascular adverse effects in conditions similar to Covid-19 infections, we do not recommend the regular NSAID use as the first line option for managing the symptoms of Covid-19 [9]. On the other hand, given the reliability of the current data, we are oriented not to modify the therapeutic protocols of non-infected chronic patients until we have new evidence indicating the opportunity or indeed the necessity to do so.

As recently stated [10], there is no time to carry on randomized controlled studies following the usual standards we are used to. In this emergency, the scientific community should leave the usual schemes, and propose instead practical research programs. This does not imply poor quality studies, but the best studies we can perform in such a short time frame, where any second is crucial to save many lives. Social separation, although effective, is only aimed to slow down the growing curve of infection: the discovery of new targeted chemicals will determine whether a patient will develop complications or make a full recovery. Similarly, the discovery of possible dangerous interactions between some classes of drugs and Covid-19 is what will prevent a further worsening of this infection in some patients, reducing the number of serious cases.

Following the invitation by EMA for timely epidemiological studies to provide adequate evidences on the effects of NSAIDS on prognosis of Covid-19 infection, our institute is about to start an epidemiologic study using our pathology registries [11]. In particular, the total joint replacement registries report data of thousands of patients, including their drug consumptions. Since Lombardy alone, a region of about 10 million people, as of today accounts for more than 51,234 positive cases (roughly 4% of the worldwide cases), we believe that our data will give specific information about the supposed risks of the use of different classes of drugs and the Covid-19 infection. Our registry records will be matched with the regional registry of Covid-19 cases to retrieve any possible correspondence between drug assumption and susceptibility to pulmonary complications. The results of the study, providing either convincing positive or negative evidence of association, will add an important piece of information to clinical care as the coronavirus pandemic continues.
